# Undergraduate Medical Research Programme: A Cross-Sectional Study of Students’ Satisfactions, Perceived Challenges, and Attitudes

**DOI:** 10.5539/gjhs.v7n5p117

**Published:** 2015-02-24

**Authors:** Alaa Althubaiti

**Affiliations:** 1 Department of Basic Medical Sciences, College of Medicine, King Saud bin Abdulaziz University for Health Sciences, Riyadh, Saudi Arabia

**Keywords:** undergraduate medical research programme, students’ perspective, satisfaction, challenges, attitudes

## Abstract

**Background:**

Implementing an undergraduate Medical Research Programme (MRP) in medical colleges may not only improve the subsequent career of medical students but also benefit the health system in general. If not designed effectively, however, such a programme could have the opposite impact. Therefore, the quality of a MRP should be evaluated continuously. This study aims to evaluate the MRP from medical students’ perspective.

**Methods:**

A cross-sectional survey study was conducted from March to April 2014 amongst undergraduate medical students at the College of Medicine, King Saud University for Health Sciences, Riyadh, Saudi Arabia. Satisfaction, perceived challenges, and attitudes towards the MRP were evaluated.

**Results:**

A total of 154 responses were collected from the students; 81(52.6%) were in the 2nd year and 73 (47.4%) were in the 3rd year of the MRP, 97(63%) were males. The mean ± SD age was 21.5 ± 0.82 years. Overall, most students were satisfied with the MRP (51.3%). The majority of students were of the opinion that there was a shortage of time to complete their work (57.6%) and a lack of motivation to do research (53.3%). Significant differences were found in the satisfaction levels and perceived challenges between students in the 2nd and 3rd year of the MRP (P≤ .013).

**Discussion:**

Assessment of medical students’ perspective towards the MRP is an important aspect of the educative process. We recommend more evaluation studies, because they ensure that programmes effectively meet their goals and continue to be improved. A solid MRP is essential and will increase the university’s profile.

## 1. Introduction

Teaching and learning methods in medical education have undoubtedly evolved over the past 50 years. This path has certainly been influenced by advances made in medical research programs that have gradually been integrated into medical curricula. The most recent teaching and learning methods include evidence-based and problem-based medicine. Both methods rely on the appropriate assessment of medical research, such as learning through elucidation and evaluation of real medical cases (Norman, 2002; Yamamoto, 1999). The synergy of medical research and education is proven to develop students’ capabilities of self-learning and abilities to tackle intellectual challenges (Fisher, 1981; Hren et al., 2004; Jacobs & Cross, 1995; Rosenblatt, Desnick, Corrigan, & Keerbs, 2006).

Recently, many universities throughout the world have integrated research into medical school curricula by establishing Medical Research Programmes (MRPs). These programmes afford medical students with research opportunities within the university. MRPs have since been a subject of numerous evaluations. Some are based on students’ opinions, while others include supervisors’ perspectives (Burgoyne, O’Flynn, & Boylan, 2010; de Oliveira, Luz, Saraiva, & Alves, 2011; Hunskaar et al., 2009; Solomon et al., 2003).

The University of King Saud for Health Sciences has incorporated an MRP into its problem-based medical curriculum from its establishment (Tamim, Al-Kadri, Zamakhshary, Al-Alwan, Al-Moamary, & Tamim, 2012). The school has been at the forefront of promoting research in the Kingdom of Saudi Arabia (KSA). The MRP is the first of its kind in the country and is directed by the College of Medicine Research Committee. The committee is comprised of expert researchers (lecturers and professors) and is supported by two full time administrative assistants. The MRP is a 4-year longitudinal programme divided into two parts (MRP I and MRP II). The main elements of the programme are presented in Table 1.

**Table 1:** Medical research programme (MRP) components

	Year	Research activity
**MRP I** (4 credited hours, final grade of 100%)	1^st^ year	- Attend research-based lectures - Choose a main supervisor (scientist, senior faculty, and/or senior hospital-based physician) and a research area - Write a learning contract (includes project title, aim/objectives, methodology, research significance, etc.) - Submit learning contract to COM-RC - Obtain revision and approval of learning contract by COM-RC
	2^nd^ year	- Attend meetings with supervisor(s) - Write a research proposal (includes the research title, duration, funds, details on materials and methods, etc.) - Attend research-based lectures with assignments on each section of the proposal. Grading on the submission and revision of the assignments is applied - Present progress presentation of research proposal orally - Submit research proposal to COM-RC - Obtain revision and approval of research proposal by COM-RC - Forward research proposal to KAIMRC for ethics approval and/or funding approval before research project initiation

**MRP II** (3 credit hours, final grade of 100%)	3^rd^ year	- Attend research-based lectures with assignments - Attend meetings with supervisor(s) - Collect and analyse all data
	4^th^ year	- Write final report (2000 – 3000 words excluding the abstract, references, and appendices). The report includes introduction/methodology/analysis/results/and conclusions. - Present final research project orally - Submit final report to COM-RC - Acquire revision and grading of final report by COM-RC

COM-RC: College of Medicine Research Committee; KAIMRC: King Abdullah Medical Research Centre.

Students have the option to conduct research independently or in groups of a maximum of four students. Students are assessed on lecture attendance, submission and revision of assignments, and presentation of their research proposals and final projects. All MRP activities are integrated into the curriculum, except the time devoted to data collection. This is considered an additional task or duty, as students are required to collect data in their spare time. A more detailed outline of the MRP and the problem-based medical curriculum in the university is provided by Tamim et al. (2012).

In the past two years, the MRP has been extensively modified and revised to integrate research-based lectures, regular meetings with supervisors, tutorials, and workshops. No studies have yet been conducted to evaluate the MRP from a student’s perspective. This study aims to evaluate the satisfaction, challenges and attitudes of undergraduate medical students toward the MRP. Understanding students’ perspectives would greatly impact its future design and management

## 2. Method

We present a cross-sectional study at the College of Medicine, King Saud University for Health Sciences, Riyadh, Saudi Arabia in which 248 undergraduate students in the 2nd and 3rd years (119 and 129 students, respectively) of the MRP were enrolled. Students in the 1st and 4th year of the MRP were excluded from this study. Not all students in the 1st year progressed through the program like 2nd and 3rd years. A number of these students were still in the process of finding a supervisor to support their research interests. Students in the 4th year had already completed an older version of the programme.

A questionnaire, based on similar surveys reported in the literature, evaluated the students’ perspectives toward the programme (de Oliveira et al., 2011; Hunskaar et al., 2009; Siemens, Punnen, Wong, & Kanji, 2010). The questionnaire included both quantitative and qualitative variables in addition to open-ended questions. Initially, the questionnaire was distributed to a pilot sample of 16 medical students to perform the reliability analysis and examine question length and clarity. Cronbach’s alpha coefficient for the scale on perceived challenges and attitudes was 0.74. Educators involved in the MRP were also requested to evaluate the questionnaire. Validation of obtained responses on satisfaction, perceived challenges, and attitudes toward the MRP used comparisons with objective measures, such as feedback from research supervisors.

The final draft was appropriately modified according to outcomes of the pilot study, and included the following items:

- demographic information (gender, year of MRP, age)
- intention to pursue postgraduate (MSc or PhD) study after medical school
- information about the research project (area of research, number of students in the project and co-supervisor(s))
- overall satisfaction with the MRP
- satisfaction with supervision provided by the research supervisor(s)
- satisfaction with MRP administration, including the organisational aspect of the programme
- perceived challenges and attitudes as closed-ended nine items in 5-point Likert scale format to address the aforementioned objective (higher agreement associated with a higher score)

Responses on satisfaction level were categorized as ‘very satisfied,’ ‘somewhat satisfied,’ ‘neither satisfied nor dissatisfied,’ ‘somewhat dissatisfied,’ and ‘very dissatisfied.’ The questionnaires were distributed over a period of approximately seven weeks (13 March 2014 until 30 April 2014) toward the end of the MRP assignments for each year. This was to guarantee that the students’ satisfaction would reflect the most recent assignments. All respondents were given information regarding study aims. Questionnaires were administered to students at the end of a regular scheduled class and each one took an average of five minutes to complete. This study was approved by the Institutional Review Board in King Abdullah International Medical Research Centre (RC14/008).

### 2.1 Statistical Analysis

The data were mainly described as means ± standard deviation (SD) for continuous variables, and percentages for categorical variables. An independent sample t-test, Mann–Whitney test, or one-way analysis of variance (ANOVA) was used to compare continuous data. To make the interpretation easier, only the combined agreement levels of ‘strongly agree’ and ‘agree’ are reported. A p-value<0.05 was considered statistically significant. Data were analyzed using the SPSS database (IBM SPSS Statistics, SPSS Inc., Chicago IL). Only a few respondents answered the open-ended questions, hence their responses were not summarised.

## 3. Results

### 3.1 Student Characteristics

The overall response rate was 62% (N=154). Response rates were 68% (N=81) from 2nd year and 56% (N=73) from 3rd year students. Characteristics of the participants are presented in Table 2.

**Table 2:** The research medical students’ characteristics

Student Characteristics	Descriptive statistics
Age, mean ± SD *y*	21.5±0.82
Gender, N(%)	
Male	97 (63)
Female	57 (37)
Programme year, N(%)	
2nd Year	81(52.6)
3rd Year	73(47.4)
Want to pursue a postgraduate study, N (%)	
Yes	152(98.7)
Area of research, N(%)	
Basic medical sciences	14(9.1)
Clinical	93(60.4)
Medical education	19(12.3)
Epidemiology and public health	28(18.2)
Other	2(1.2)
More than one medical student in the research project, N (%)	85(55.2)
Co-supervisor(s), N (%)	
Yes	64(41.6)

Total number of participants = 154.

The mean±SD age of respondents was 21.5±0.82 years, and 97 (63%) were male. Most students were conducting ‘clinical’ research (N= 93, 60.4%) as their research subject area. This was followed by ‘epidemiology and public health’ (N=28, 18.2%) and ‘basic medical sciences’ (N=14, 9.1%). The majority of the students conducted research in groups of 3 or 4 students (N=85, 55.2%) under the direction of one main supervisor (N=90, 58.4%).

### 3.2 Satisfaction

The majority of students were ‘somewhat satisfied’ (N=58, 37.7%) or ‘neither satisfied nor dissatisfied’ (N=36, 23.4%) with the MRP. Students were ‘somewhat satisfied’ (N=75, 48.7%) or ‘very satisfied’ (N=30, 19.5%) with the supervision provided by the research supervisor(s). Students were also ‘somewhat satisfied’ (N=62, 40.3%) or ‘very satisfied’ (N=42, 27.3%) with the administrative support provided by the programme.

Significant differences were found in satisfaction levels between students in the 2nd and 3rd year of the MRP. Students in the 2nd year of the programme (mean score 1.96, SD=1.16) were less satisfied with the programme (mean score 2.75, SD=0.91; P< .001), supervisor(s) (2nd year: 2.44±1.18, 3rd year: 2.88±0.96; P< .013), and administrative support (2nd year: 2.68±0.77, 3rd year: 3.11±0.95; P= .002), compared to 3rd year students. No significant differences were found among other demographic characteristics with regard to satisfaction level. Details of the results are presented in Table 3.

**Table 3:** Satisfaction levels amongst the medical students with the overall MRP, supervisor(s) and administrative supports

	Very satisfied N(%)	Somewhat satisfied N(%)	Neither satisfied nor dissatisfied N(%)	Somewhat dissatisfied N(%)	Very dissatisfied N(%)	*P* value*
**Overall programme**						
2^nd^ Year (N=81)	6(3.9)	25(16.2)	19(12.3)	22(14.3)	9(5.8)	<.001
3^rd^ Year (N=73)	15(9.7)	33(21.4)	17(11.0)	8(5.2)	0	
All students	21(13.6)	58(37.7)	36(23.4)	30(19.5)	9(5.8)		
**Supervisor(s)**						
2^nd^ Year (N=81)	14(9.1)	34(22.1)	13(8.4)	14(9.1)	6(3.9)	.013
3^rd^ Year (N=73)	16(10.4)	41(26.6)	11(7.1)	1(0.6)	4(2.6)	
All students	30(19.5)	75(48.7)	24(15.6)	15(9.7)	10(6.5)		
**Administrative supports**						
2^nd^ Year (N=81)	10(6.5)	40(26)	26(16.9)	5(3.2)	0	.002
3^rd^ Year (N=73)	32(20.8)	22(14.3)	14(9.1)	5(3.2)	0	
All students	42(27.3)	62(40.3)	40(26.0)	19(6.5)	0	

Total number of participants = 154.

* Independent sample t-test.

### 3.3 Perceived Challenges and Attitudes

The results related to students’ perceived challenges and attitudes, which are summarized in Table 4. The main perceived challenges were: a shortage of time to complete work (N=88, 57.6%), a lack of motivation to do research (N=81, 53.3%), and supervisor(s) were not readily available to help deal with key challenges in the MRP (N=79, 52.0%).

**Table 4:** Agreement levels amongst the medical students on the perceived challenges and attitudes towards the MRP

	2^nd^ Year (N=81)	3^rd^ Year (N=73)	All students	*P* value*

	Agree N(%)	Agree N(%)	Agree N(%)	
1. Shortage of time to complete work	42(27.5)	46(30.1)	88(57.6)	.007
2. Shortage of funds	4(2.7)	14(9.4)	18(12.1)	.001
3. The length of the medical research programme is adequate	22(14.8)	37(24.8)	59(39.6)	< .001
4. Lack of library resources	31(21.0)	36(23.7)	67(44.7)	.451
5. Lack of motivation to do research	48(31.6)	33(21.7)	81(53.3)	.345
6. Lack of adequate knowledge in the area of research	30(20.0)	37(24.7)	67(44.7)	< .001
7. Research supervisor(s) provide good guidance and support	32(20.9)	44(28.8)	76(49.7)	< .001
8. Research supervisor(s) are not readily available to help deal with key challenges	53(34.9)	26(17.1)	79(52.0)	< .001
9. Family problems and other commitments	24(15.9)	17(11.3)	41(27.2)	.628

Total number of participants = 154. Only agreement levels are reported for easier interpretation. Both agree (score=3) and strongly agree (score=4) were combined.

* Mann-Whitney test between responses of 5-point Likert scale.

Significant differences were found between students in the 2nd and 3rd year of the MRP with regard to perceived challenges and attitudes. Students in the 2nd year noted that unavailability of research supervisor(s) (34.9% vs. 17.1%; P< .001) as a greater challenge than those noted by 3rd year students. Students in 3rd year, however, considered that a lack of time to complete work (30.1% vs. 27.5; P= .007), shortage of funds (9.4% vs. 2.7%; P= .001), and lack of adequate knowledge in the area of research (24.7% vs. 20.0%; P<.001) were all the vital shortcomings of the MRP programme. These students noted that the length of the medical research programme was adequate (24.8% vs. 14.8%; P< .001), and that their research supervisor(s) provided enough guidance and support (28.8% vs. 20.9%; P< .001). No significant differences were found amongst students with respect to other demographic characteristics.

## 4. Discussion

Assessment of medical students’ perspectives towards the MRP is an imperative aspect of the educative process. To our knowledge, this study is the first of its kind to assess an MRP at a university in KSA. Two groups of medical students in both the 2nd and 3rd year were enrolled. Students in the 3rd year were in the clinical phase of the undergraduate curriculum and were required to attend hospital rounds. Therefore, their lecture attendance at the time of data collection was slightly low, which explains the difference in response rates. The study revealed several characteristics of research practice amongst the medical students. Moreover, results showed that students’ satisfaction, perceived challenges, and attitudes toward the MRP differed between the 2nd and 3rd years.

The majority of undergraduate students desired to pursue postgraduate degrees. This result substantiated earlier studies in the United States and Norway (Hunskaar et al., 2009; Solomon et al., 2003). Implementing research into the undergraduate curriculum may increase students’ motivation to pursue a postgraduate degree. It should be noted that the number of medical graduates is increasing in KSA. This should be met with a concomitant increase in educational opportunities for prospective postgraduate medical students (Althubaiti & Alkhazim, 2014).

As anticipated, most students preferred clinically oriented projects–a result similar to that reported by Zier, Friedman, and Smith (2006). These projects are mainly retrospective and data collection is based on chart reviews or questionnaires. Students may not be aware of other types of research and how they can relate to their field, and thus more effort to encourage students to work outside their fields or in interdisciplinary research should be promoted. These opportunities can broaden knowledge and increase competiveness and self-confidence.

Overall, the majority of students were satisfied with the MRP. Students in the 2nd year appear to be less satisfied than 3rd year students, and have adopted a more negative attitude toward the programme. This could be due to the numerous tasks students perform to submit well-written research proposals at the end of the year, including extensive literature reviews. Students in the 3rd year, however, already had developed confidence in their research skills and in the MRP. Thus, advancement in the MRP may correlate to satisfaction with the programme. These findings confirm results of an earlier study, in which a more positive attitude toward research courses in medicine correlated to more advance students (Vujaklija et al., 2010)

Students in the 2nd and 3rd year agreed on a number of challenges. These were very similar to those identified in previous studies (de Oliveira et al., 2011; Hunskaar et al., 2009; Siemens et al., 2010). Two of the main challenges were unavailable research supervisors and inadequate time (de Oliveira et al., 2011; Siemens et al., 2010).

More 2nd year students reported that their supervisors were not readily available to help with key challenges in the MRP. They were more dissatisfied by their guidance and support than 3rd year students. A possible explanation is that these students require more support than 3rd year students. Most supervisors are senior hospital-based physicians and not full-time faculty members. Thus, their time to meet with students may be limited. Different strategies can circumvent these challenges. One strategy could involve assigning an academic advisor or a co-supervisor to students. This supervisor should be a full time faculty member to provide extra support and feedback. A second strategy would be to provide supervisors with incentives and recognition, like the “best supervisor” award. A third strategy could introduce a protected time initiative so supervisors spend more time with their students. Lastly, establishing supervisory training courses would enhance mentoring and guidance skills. The previously mentioned strategies could even facilitate supervisors to partake in these courses.

Students in their 3rd year agreed more than 2nd year students on a shortage of time to complete their work. This is reasonable, considering their increased clinical duties. They also noted that a shortage of funds was a challenge. Often, funds take a relatively long time to be approved, which does not take research time for students into consideration. For example, some students could not even begin research due to pending grant approval. Students in their 3rd year also indicated that they did not sufficiently understand their chosen research project. This was rather surprising, since by MRP II (Table 1), students should have detailed understanding of their research. Medical supervisors and teachers play the most important roles. The approach by which background information is taught to students must be reassessed to positively impact research experiences (Kilminster & Jolly, 2000). Different studies have investigated this matter, but they are beyond the scope of this paper.

In connection with the identified challenges, a significant number of students indicated they lack motivation to perform research. The reasons could not be extrapolated from this study. Arguably, these feelings could arise from documented challenges. Future studies should investigate this issue thoroughly.

This study only assessed the MRP from students’ perspectives; yet it is important to consider that programme productivity rests on students’ performances. Thus, monitoring performance by tracking posters and publishes results, may enhance research skills, competitiveness, and motivation. A major limitation of this study is that it does not provide an overall evaluation of the programme.

## 5. Conclusion

In conclusion, the vast majority of the surveyed undergraduate medical students were satisfied with the MRP. However, a few challenges and attitudes towards the programme were identified. We recommended a number of solutions to overcome these challenges and improve the medical students undergraduate research experience.

It is crucial to stress the importance of programme evaluation studies as the present one, because they ensure that programmes effectively meet their goals and continue to be improved. After all, a sound MRP is essential and will without doubt increase the university’s profile, both nationally and internationally.
